# The body in Martin Amis’s *Experience* (2000)

**DOI:** 10.1080/0950236X.2016.1237986

**Published:** 2016-11-24

**Authors:** Neil Vickers

**Affiliations:** ^a^ Department of English Literature, King's College London, London, UK

**Keywords:** Martin Amis, psychosomatics, psychoanalysis, phenomenology, mind-body

## Abstract

This paper focuses on the presentation of the body in Martin Amis’s memoir *Experience* (2000) and compares Amis’s account of the growth of his mind and body with ideas put forward by writers in the phenomenological and psychoanalytic traditions. Using the ideas of body schema, projective identification and idea of safety, it advances a new conceptual framework for the thinking about the contribution that the body makes to selfhood in autobiography.

This paper puts forward a reading of Martin Amis’s memoir, *Experience* (2000).^1^ Surprisingly little has been written about this virtuosic book. It features in two monograph studies of Amis’s fiction, chiefly as a source of biographical information about Amis and his family.^2^ But I shall argue that, quite apart from its documentary value, it has special relevance for theorists of life-writing and for anyone with an interest in the autobiographical representation of embodiment. For *Experience* makes some striking claims about what it means to have a mind and a body. Some of these are explicit. Amis tells us, for instance, that he had neglected his teeth for decades because, unconsciously, he believed that losing them would enable him to effect a deep change in his personality at some point in the future.

A good plan would have been to keep on going to the dentist. My respect for the unconscious continues to grow. My unconscious mind might not have thought much of the plan either, but it worked around that and made its preparations. Really, the conscious mind can afford to give itself a rest. The big jobs are done by the unconscious. The unconscious does it all.^3^

Teeth – his own or other people’s – were often on Amis’s mind whenever he found himself in emotionally charged situations. The first time he saw his father after the breakdown of his parents’ marriage, Kingsley was sporting a new set of teeth, having generally avoided smiling in public until then. The new teeth were a sign that his father had taken on a new identity. When his mother Hilary had hers removed some years later, Amis was ‘harrowed’ by this vision of a ‘parody mother’, reminded once more of the separation and all the sadness it had brought with it. His decision to travel to New York in 1994 to have his teeth replaced by implants was a response to a complex personal crisis. His first marriage was disintegrating and he had recently learned that his cousin Lucy Partington, who had gone missing in December 1973, had been murdered by the serial killer, Fred West. Kingsley was becoming increasingly ill and probably would not live much longer. Losing his teeth was a rite of passage enabling marriage to his second wife, Isabel Fonseca, and the acknowledgment of a daughter, Delilah Seale, whose existence had been revealed to him in 1977 but whom he had never known. It also set in motion a process by which he came to terms with Kingsley’s death and found a new father-figure in Saul Bellow.

All of this will be familiar to any reader of *Experience*. I want to suggest that the memoir also contains a less explicit set of claims about what it means to have a mind and a body. It is less explicit because it belongs to the things the book shows rather than those it tells and it is never summed up into propositional statements by Amis. But it can be condensed into propositional form. Its leading claims are as follows. People are first and foremost *bodies*. People who are important to us are bodies with known psychophysical trajectories. Only at the most sophisticated level of our experience do we take account of a person’s humanity. Amis’s novel *The Pregnant Widow* (2011) begins with an epigraph taken from Ovid: ‘Now I am ready to tell how bodies are changed / Into different bodies’.^4^ I will suggest that the episodes in *Experience* are best read as attempts by the author to change his body into a succession of different bodies. Amis describes his adolescence as a progress from a highly disorganised mind-body, acutely vulnerable to physical attack, to a less disorganised one, characterised by an improved ability to register and withstand more of his own bodily experience. Improvement comes about as a result of his recognition of other people. Crucially, such recognition begins with identification with their *physical* experience. It is only when we open ourselves up to other people’s physical experience that we can know what it is like to be them. Intercorporeality, in other words, must precede intersubjectivity. To share a phenomenal world with someone else, you have to be willing to take their physical experience as a template for your own and *vice versa*.

Many aspects of my argument have a bearing on Amis’s fiction. He has often been criticised as a novelist for depriving his characters of complexity. As his fellow-novelist John Banville has put it:

The comic energy never flags, the metaphors dazzle, and whether he is describing a dog defecating or the play of light on a stretch of the Thames he achieves an intensity of poetic specificity on a level with the work of such masters of style as Nabokov and Updike. In the manner of character and plot, however, there is overall a particular haziness, a lack of or withholding of focus, which can leave the reader feeling baffled and slightly cheated. Even the main figures in the novels, John Self in *Money*—“I’m called John Self. But then who isn’t?”—Guy Clinch and the talentless Keith Talent in *London Fields*, and the rival writers Richard Tull and Gwyn Barry in *The Information*, seem not so much portraits of plausible human beings as marionettes gesticulating wildly in the glare of Amis’s pyrotechnical prose. The women characters in particular can seem thin to the point of two-dimensionality … ^5^

These are powerful points, well made. But if *Experience* has anything to tell us about Amis as novelist, it is that he is most fascinated by those aspects of personhood that subtend our capacity for moral experience. These things are primitive and pre-moral. Growing up involves learning to cope with a wide range of bodily discomforts. Such discomforts are part of what we might call psychosomatic normality. Adapting to the rhythms of one’s psychosomatic reality is a productive process that enables the *self* to develop, not just the body. This is the process that Amis tries to capture in his memoir. It may be that in his novels he is concerned with the other side of the coin, that is to say, with characters who do not develop because they are unable to form what the psychoanalyst Donald Winnicott memorably called ‘a psychosomatic partnership’.^6^ In a very fine paper published in this journal, Philip Tew makes precisely this point in relation to *Money*.^7^ Tew argues that the novel’s most fundamental subject is what it means to live in an ‘alexithymic’ world. Alexithymia is a disorder in which individuals have difficulty finding words to describe their emotions. Instead, they somatise their emotions.^8^ Tew observes that the hero of *Money*, John Self, tries to live his life by denying human inter-relatedness. He is ‘sexually compelled by women, but is also repulsed by their physical demands and presence’.^9^ He is obsessed by money and pornography. He likes his English girlfriend for her ‘brothelly know-how and her top-dollar underwear’ and he talks to her about money during sex.^10^ But he has no interiority as such. Amis is no John Self but he is candid about the defensive, one might say ‘alexithymic’ purposes served by his dental obsessions. The last time he saw his friend Lamorna Seale and she told him he was the father of her daughter Delilah and gave him a photograph of her, he was ‘as usual obsessively alert to the health and prettiness of [Lamorna’s] dentition’.^11^ After showing his mother the photograph (Without looking up, she said ‘*Definitely*.’), Delilah became an absence in his life, one of the two girls whom he designates ‘My Missing’ (Lamorna committed suicide the following year). The other is his cousin Lucy. Nine months after her disappearance, Amis went to stay with his mother who was also being visited by Lucy’s mother:

They were over by the draining board, preparing a hot drink, while I remained at the table, deep in an unpleasant, unconstructive and above all familiar dental reverie; a recent explosion in the top deck had made the right-hand side of my naval cleft tender to the touch – and so of course I kept touching it, feeling, testing it … I woke up when I realised that the two sisters, for the first time in my presence, were talking about Lucy.^12^

The implication is that toothache deadened him to at least part of their conversation. I could cite many more instances of this alexithymic mode of functioning. But *Experience* is actually an account of a belated emergence *out* of alexithymia: through intense physical pain, into mourning, culminating in a new sense of relationship with others.

John Self’s undoing takes the form of a physical and psychological collapse. Significantly for my purposes, it begins with intense toothache.

My head is a city, and various pains have now taken up residence in various parts of my face. A gum-and-bone ache has launched a cooperative on my upper west side. Across the park, neuralgia has rented a duplex in my fashionable east seventies. Downtown, my chin throbs with lofts of jaw-loss. As for my brain, my hundreds, it’s Harlem up there, expanding in the summer fires. It boils and swells. One day soon it is going to burst.^13^

Tew believes that the alexithymic world of *Money* ‘expresses something of the disorder characterised by Gilles Deleuze and Félix Guattari in *Anti-Oedipus: Capitalism and Schizophrenia*. In his view, Self’s schizoid existence is ‘diagnostically suggestive of wider, negative cultural tendencies’. It reflects ‘the propensities inculcated by an era of commodified consumption, excess profit, and aggressive egoism’.^14^ Although I find this argument compelling, I will not be able to consider the wider political implications of *Experience* in any depth, even though there is a great deal to say about them.^15^ Neither will I be able to do full justice to the literary virtuosity of Amis’s memoir. Instead I will attempt to explain the dynamics of Amis’s metamorphoses from one body into another. To do this, I will make three claims using theoretical concepts derived from phenomenology and psychoanalysis.

First, I will argue that Amis’s recovery from alexithymia turns on his discovery that he has always shared a body schema with other people. The idea of the body schema I take from Paul Schilder (1886–1940 who is responsible for the widely-used distinction between *body image* and *body schema*).^16^ The body schema is the sum of the mind’s representations of where particular body parts are in relation to the outside world and in relation to one another. When we feel well, we are barely conscious of it. The body image, by contrast, is a way of representing to ourselves how our body must appear to others. Schilder thought that the most decisive factor in the formation of a satisfactory body image was the feeling of being loved by others. This enables us to love our own bodies which in turn has a beneficial effect on the body schema. ‘Feeling our body intact is not a matter of course’ he wrote. ‘It is the effect of self love. When destructive tendencies go on, the body is spread over the world’.^17^ Although Schilder was very alive to the social dimensions of bodily experience, he says little about the uses to which we put other people’s body schemas. But I think that the introjection and projection of body schemas is one of the central features of Amis’s memoir.

I ground this process in Melanie Klein’s idea of projective identification, which constitutes my second major theoretical concept.^18^ Klein claimed that there is a universal fantasy common to all human beings that we contain within our bodies, *concretely and immediately*, people and bits of people with whom we have emotional relationships. She called these introjects ‘internal objects’.^19^ They people our ‘inner world’. At its most basic, projective identification is a fantasy of a special kind in which part of the self is felt to be located in another person. This gives us a concrete and immediate existence in other people’s bodies. Projective identification has many uses. It provides a means by which we can rid ourselves of intolerable experiences. A baby for instance might project his distress into his mother. Significantly Klein emphasises that sometimes good experiences are projected. Projective identification is also a primitive way of getting to know other people. As Klein put it in 1959, ‘By attributing part of our feelings to the other person, we understand their feelings, needs and satisfactions’.^20^ In considering any single instance of projective identification, it is often helpful to ask whether its primary goal is to take possession of something in someone else or to get another person to carry some unwanted aspect of ourselves. Ronald Britton calls the first sort of projective identification acquisitive and the second sort attributive.^21^ Perhaps the feature that distinguishes projective identification from Freudian projection is its assumption that we can never simply disown something in ourselves; we always take on aspects of others in the process of doing so. All projection is somewhat introjective and all introjection is somewhat projective. Klein’s collaborator, W. R. Bion, thought that young infants use their mothers as auxiliary minds.^22^ A baby cries because he is overwhelmed by a fear of death. He cries in order to have his mother take this unbearable feeling into her own mind and transform it so that it can be returned to him in a form he can tolerate. The transformation of intolerable unmediated physical experiences into thoughts was made possible by a specific introjective capacity in the mother which he called reverie. I will suggest later that Amis turns to others as containers for his psychophysical distress.

Projective identification attempts to locate our experience in other people’s bodies. Usually, we are too caught up in the meanings of our dealings with others to pay much attention to this fact. But in the case of the experiences described in Amis’s memoir, the bodily nature of projective and introjective processes is foregrounded to a quite exceptional degree. Although he may not have known about the theory of projective identification, the French phenomenologist Maurice Merleau-Ponty’s attempt to understand the lived body in terms of its practical engagement with the world has much in common with Klein’s projective–introjective model of psychic life. ‘I experience my own body as the power of adopting certain forms of behaviour and a certain world’, he writes in *The Phenomenology of Perception*.^23^ So, for example, when I see someone else play the piano I experience what I witness as a set of suggestions to my own motility. Maybe if I did those things, I would make beautiful sounds too? As Merleau-Ponty puts it in *The Phenomenology of Perception*:

now, it is precisely my body that perceives the body of another, and discovers in that other body a miraculous prolongation of my own intentions, a familiar way of dealing with the world. … [T]he anonymous existence of which my body is the ever-renewed trace henceforth inhabits both bodies simultaneously.^24^

In ‘The Child’s Relations With Others’ – notes based on a series of lectures he gave in 1951, in which he mentions Klein’s work with children – Merleau-Ponty explains how this comes about. We take our cues from other bodies by means of a body schema. The behaviours I witness of other bodies in the world act on my body schema.

Husserl said that the perception of others is like a “phenomenon of coupling” *[accouplement].*The term is anything but a metaphor. In perceiving the other, my body and his are coupled, resulting in a sort of action which pairs them *[action à deux].* This conduct which I am able only to see, I live somehow from a distance. I make it mine; I recover *[reprendre]* it or comprehend it. Reciprocally I know that the gestures I make myself can be the objects of another’s intention. It is this transfer of my intentions to the other’s body and of his intentions to my own, my alienation of the other and his alienation of me, that makes possible the perception of others.^25^

In the depths of our minds, it was not some other person playing the piano at all; it was us. What I find so helpful in Merleau-Ponty’s account of child development is the claim that in order to have a clear sense of my own body schema I have to lay hold of someone else’s. I believe that this process can be seen to operate again and again in Amis’s memoir.

The third theoretical concept that I shall draw upon is the psychoanalyst Joseph Sandler’s notion of ‘safety’.^26^ Sandler thought that in addition to avoiding anxiety, human beings in general and children in particular often seek to maximise a sense of safety or security. Safety in Sandler’s sense is not a form of excitement and – strikingly, in a psychoanalytic paper – Sandler says that the pursuit of safety is often more important than the pursuit of excitement. Now plainly, illness undermines the sense of safety, sometimes catastrophically. But having a strong sense of safety can make illness easier to bear. Sandler thought that the sense of safety was rooted in ordinary perceptions. By making sense of the world perceptually, we make it our own. The importance of the notion of safety for the argument I am about to make is that it represents a counter-traumatic force in the personality. I will suggest, moreover, that having a strong sense of safety enables us to contain others’ experiences in Bion’s sense of transforming them into something thinkable. The visceral sensation of danger is replaced by a mental one of understanding.

## The structure of *Experience*

When *Experience* first appeared, it was one of only a handful of memoirs by novelists that was not primarily about the making of the novelist or the novels. In a perceptive early review of the book, John Lanchester contrasted novelists’ memoirs that made conspicuous use of fictional devices with those that ‘let life have its messiness, and let the book pay the necessary price in terms of formal imperfection’.^27^ Nabokov’s *Speak, Memory* was the model of the artful memoir while the four volumes of Anthony Powell’s diaries exemplified ‘life-over-art’. ‘All novelist’s memoirs’, Lanchester went on, ‘exist somewhere on this Nabokov-Powell continuum’.^28^ Inexplicably, Lanchester found *Experience* to be ‘well towards the life-over-art end of the spectrum’. Yet Amis’s book abounds in novelistic tricks. Anyone who has read it right through will be able to recognise all sorts of significance in apparently innocuous details in the opening chapter alone, such as the fact that the two girls in the photographs Amis keeps on his desk are smiling. And the reader coming to the book for the first time will probably sense from the artfulness of the presentation that they are being entrusted with secrets, even if they do not yet know what the secrets are or why they matter. Before long, we are confronted with regular use of intertexts, *mises-en-abîmes*, and symbols and this makes us wonder about the author’s sense of the scale of his work. The use of Joycean punctuation – em-dashes at the beginning of quotations with no mark to signal an ending – suggests it might be vast: on a par with *Ulysses*, say.

Perhaps the most important point to make about *Experience* is that its autobiographical ambitions are unusual. It is not a chronicle. It is a record of how Amis’s past was transformed by the events of 1994–95. Amis appears to flit from memory to memory, without much regard for chronological sequence, as if he were free-associating. At the same time, the fact that the material is organised into chapters suggests that the arrangement of the scenes will eventually be revealed as meaningful.

In the early chapters of the memoir, Amis calls to mind occasions when he found his bodily experience overwhelming and even annihilating. These are interspersed with recollections of moments in which his body did not interfere too much with a good experience. But such moments are presented as having been comparatively rare. Here is an example taken from the second chapter, entitled ‘Rank’. ‘Rank’ is vague about precise sequence but it appears to begin in Amis’s bedroom. Amis, in his late teens, is sunk in ‘a bottomless adolescent *cafard*’ ‘taking an entire day to transport a single sock from one end of [his] bedroom to the other’, unable or at any rate disinclined to concentrate, playing truant from school in order to enjoy the company of his friend Rob:

betting in betting shops (not the horses: the dogs), mincing up and down the King’s Road in skintight velves and grimy silk scarves and haunting a coffee bar called The Picasso, and smoking hash (then £8 an ounce) and trying to pick up girls.

Rob, like Amis, is small in stature and diffident. ‘I always feel such a short-arse in the Picasso’, he explains. These excursions often ended with the two friends going home in fright and ‘smoking [themselves] into a state of clinical paranoia’. Other adventures mentioned include mixing with ‘the giantesses of the gentry’ and feeling ‘as if we were walking between everybody else’s legs’.^29^ ‘Rank’ is about adolescents at sea in the world and full of self-loathing. Their bodies are a source of shame and pain to them. They do not believe they are entitled to anyone’s respect; they are ‘rank’ in the sense described by *OED* as ‘Highly offensive or loathsome, esp. morally; evil, abominable, foul’. Shortly after these experiences, Amis leaves London for Sussex Tutors, a boarding crammer. Sussex Tutors has much in common with his bedroom. It is ‘a ramshackle warren that seemed to be all attic … it was said that the building had once been a nursing home’ and it was surrounded by nursing homes in a city that was itself one vast nursing home.^30^ Amis feels like a convalescent. And ‘now at least, I was in love with literature. I read poetry and I wrote poetry’. The central opposition of this chapter is between a body that is overwhelmed by its own inadequacy, especially when faced with the external world and one that is ‘nursed’ and allowed to be alone. The somatic feeling that links the events of the first half of the chapter is humiliation at being seen. The somatic feeling that dominates the second half is being able to look without fear. Sussex Tutors, a geriatric institution in disguise, can bear his contempt, as can the town’s inhabitants. The young Amis looks on Brighton as ‘a town arranged like rows of seats around a stage, the sea’. He reads. And perhaps he looks on himself in a new way through his adoption of the name ‘Osric’, Claudius’s spy in *Hamlet*. He is in Elsinore, albeit in an ignoble station. Here at least are the beginnings of self-respect.

Merleau-Ponty famously defined the body in terms of its potential. I *am* my body and my body is the potentiality of any given world.^31^ It allows us to ‘reckon with the possible’.^32^ In a similar fashion, the fear and shame that grip Amis as he ‘minced’ up and down the King’s Road with his friend Rob might be seen as by-products of an attempt to ‘reckon with the possible’. The friends have a shared body schema. They both feel that they are seen as small, frightened and effeminate. Using the theory of projective identification, we might suggest that they project their own self-loathing into the other people on the street and as a result find it is everywhere. In the second vignette, looking is a less dangerous activity. The elderly in their warrens are less ready to pass judgment than the modish habitués of the Picasso. Looking back, he says he felt himself to be ‘a gilded and a repulsive creature’.^33^ It was better than being merely repulsive.

In these early chapters, the characteristic movement is from extreme physical awkwardness (intensified by feelings of shame, loneliness, and anxiety), to brief self-acceptance, culminating in the consciousness of another person’s suffering. In a virtuosic touch, this consciousness is hidden from the reader in the first couple of chapters – a point to which I shall return. But when it does appear, starting in chapter 3, Amis depicts himself as being unable to do much with it; indeed it often makes him more diffident. Here is how he describes his life in London in 1974, not long after the publication of his first novel:

My headaches and faceaches (and my vestigially sebum-rich complexion) made me feel like a student. The high principles, or essential indifference, of the girl I was futilely orbiting (kisses, nothing more), made me feel like a student. At the same time, although I was edging forward into it, the adult world of promotion and preferment still looked alien and menacing to me. There was still the suspicion, despite all the current evidence, that you would not only fail but actually go under. Perhaps everybody has this. Christopher Hitchens had it: we called it ‘tramp dread’. Earls Court was certainly very fully furnished with tramps, drunks, beggars, babblers. Again in the mansion flat itself there was an old doctor, close to retirement, who sometimes showed up for the night; I would see him slumped over a sherry bottle in the lino-draped kitchen, or staggering and flailing around in a beltless bathrobe and unbelievable Y-fronts (shapelessly swilling [sic], and mackerel-grey).^34^

In this example, the down and outs of Earls Court are credited with a power to absorb Amis and Hitchens into their own bodies (with all the attendant troubles of those bodies) while the adult world of promotion and preferment exercises a power of repulsion. He and Hitchens identify with one another (through their body schema) and project their shame and self-doubt into the down-and-outs of Earls Court in order to reinforce the part of them that belongs in the adult world of promotion and preferment. But the figure of the alcoholic old doctor ‘close to retirement’ (i.e. still professionally active) but with little grip on his own dignity suggests that this projection will only get them so far. In one of the earliest and still most-cited accounts of Klein’s hypothesis, Arthur Malin and James Grotstein stressed that the redistribution of characteristics effected by projective identification does not always work to the advantage of the projector. ‘The external object now receives the projected parts, and then this alloy – external object plus newly arrived projected part – is re-introjected to complete the cycle’.^35^ The old doctor is a perfect example of a re-introjected hybrid of external object plus projected part. He is no more a tramp than Amis or Hitchens but his slumping, staggering and flailing makes him a container for their deepest fears.

The next chapter, ‘Learning About Time’, comprises six episodes, four of which are set in Spain. The first takes place in 1962 when Amis’s family visited Robert Graves and his wife at their home in Majorca. The couple spent an evening looking after the Amis children while Kingsley and Hilary went out. To pass the time, Graves suggested they compose a poem together.

And when Graves said, ‘Philip. Why don’t you begin?’, my feared, revered and much adored brother instantly and typically reached for the most subversive – and above all the nearest – thing to hand. ‘There was an old farmer who sat on a rick … ’ My ears hummed: we’re for it now, I thought … Graves smiled, and, glancing downwards, said lightly, ‘You’re not meant to know that poem.’ I think it was Beryl who got us going on something about domestic animals.^36^

The second incident, which also takes place at Graves’ home in Majorca, occurred six years later. Before going to university Amis drove to Spain with three friends, including Rob.

Then Rob said to Robert,
  - Make that mountain open up.
  - What?
  - Turn it into a volcano.
  - What?
  - Go on. You can do it. Make that cloud go away.
  - Oh, you’re –
  - Summon a tidal wave.
  - You little –
  - Make the moon come out.
  - Ooh, you –
  - Make the –
And Robert got hold of Rob and roughly tickled him.
A couple of hours later the Mini Moke was edging its way down the drive. Graves kept running back to the house to bring us more fresh-baked bread, more labelled jars of pickles and homemade jam.^37^

These two episodes turn on an older person responding kindly to a younger person. Graves understands that the young need their elders’ kindness and forbearance and charity (the gifts that Graves presses on Amis and his friends as they leave). The result is not gratitude so much as a sense of safety. Safety, not time, is the true theme of ‘Learning About Time’. It is first and foremost a physical feeling but is also an essential component of psychological wellbeing. It is worth noting that all of the anecdotes Amis relates concerning safety highlight its emergent, intermittent character. It comes and it goes; but eventually it establishes itself.

Its absence is explored in the next memory. Shortly after leaving Graves for the second time, Amis and Rob begin their journey back to London. When their car breaks down in a small town in Southern France, they phone home for money so that they can have it repaired. Family members suggest they get a job but the two friends cannot countenance that possibility and instead ‘[settle] down to a week of patient trembling and starving and hanging around the post office’ until the money comes through.^38^ They reclaim their car and with their remaining 15 francs, buy strikingly childish provisions: ‘some glacier-mints, some coffee-cream biscuits and some orangeade’.^39^ The neck of the orangeade bottle snaps off as Amis tries to open it, ‘dramatically gashing my hand’.^40^ And when his turn comes to take the wheel, he almost crashes into an ‘oncoming pantechnicon’ when someone throws a cigarette down the back of his jeans. It is tempting to speculate that these events were not just random instances of bad luck but a means of expressing how impaired their sense of safety had become. This part of Amis’s memoir ends with oblique allusions to Rob’s subsequent difficulties in life, ‘his ordeals of park bench, of winter coalhole, or shelterlessness, and prison’ (‘an eight-month sentence for a domino effect of drunk-driving offences’).^41^ Tramp dread, indeed. Rob, it seems, never managed to create a sense of safety in himself.

All of the remaining episodes in this chapter are about attempts to develop one’s sense of safety by learning to withstand physical insults of various kinds. The first vignette takes place in Andalucia in 1974 where Amis’s mother Hilary has gone to live with her third husband and their young son Jaime. One day Hilary becomes ‘the victim of an irresolute sexual assault by a local youth’. She screams at him ‘*Venga! Venga!*’ believing it meant ‘Go away! Go away!’ when it actually meant ‘Come on! Come on!’^42^ She should have said *Fuera! Fuera!* In spite of this experience, Spain remains the country Hilary loved best. Her reason becomes apparent to her son a few days later when the two walk down a crowded street and notices Rafael, ‘a spectacular spastic with an unbelievable gait’.

As Rafael, a flailing blur, inched along, and as passersby greeted him with cries of *Eh, coño!* and an embrace and a mock left hook, my mother turned to me and said, - I love living in Spain. I now regard him as *completely* normal.^43^

Watching Rafael walk down the street he realises it is possible to be awkward without being ashamed. Indeed, it is even possible to be awkward and to feel liked. As he watches others in this remarkable situation, he starts to see them as versions of himself. Again, using the theory of projective identification, we might say that Amis identifies with everybody else on the street by projecting the part of himself who is ashamed of being small and having bad teeth into Rafael. But he also identifies with the crowd in recognising how easy it is to use Rafael for this purpose. By acknowledging this, the crowd give Rafael a kind of love. (This is surely what Amis’s mother means when she says ‘I love living in Spain. I now regard him as *completely* normal’.) For the first time in the memoir, Amis can reflect upon what it means to share a body schema with someone else.

In Spain, people were openly unashamed of having bad teeth. Spain was a ‘place that had yet to experience dental self-consciousness’, and it ‘suited me down to the ground because I hadn’t smiled unreservedly for five years’.^44^ It was a safer place. The last two memories are the alexithymic ones I have already touched upon. They detail his final meeting with Lamorna Seale, who ‘never seemed stronger or happier’ (an impression strengthened by her healthy and pretty dentition) and his aunt’s visit to his mother in which he awakes from his dental reverie to hear her say ‘in a steady and unemphatic voice that not a minute passed without her thinking of Lucy and wondering where she was’.^45^

In a paper published in 1949, and against the grain of prevailing psychoanalytic thinking, Donald Winnicott suggested that some somatoform disorders should be seen not as acts of repression so much as primitive attempts at mentalisation. They represent the ego’s first attempt to register a difficulty.^46^ They do not tell a story in the way that say Freud’s patient Dora’s limp tells a story (that she had, for instance, taken a ‘false step’ when she rejected Herr K). Winnicott was led to this view when he tried to place psychosomatic disorders in a developmental framework. Under the influence of Klein and Anna Freud, child development supplied the framework for most debates inside the British Psychoanalytic Society in the 1940s and long afterwards. Winnicott, who was at that time associated with the Klein group, took as his starting point the idea that the infant psyche begins as nothing more than a mechanism for the imaginative elaboration of somatic experiences. The infant finds that his bodily sensations are a source of pleasure or unpleasure. But ‘the psyche is not felt by the individual to be localized in the brain or indeed to be localized anywhere’. As he develops, he finds that his capacities for reflection and fantasy become a source of strength – but only if his ‘holding environment’ is ‘good enough’. For ‘Certain kinds of failure on the part of the mother, especially erratic behaviour, produce over-activity of the mental functioning’.^47^ This causes the child to mother himself. Non-hysterical somatic disorders are attempts to

draw the psyche from the mind back to its original intimate association with the soma … One has also to be able to see the positive value of the somatic disturbance in its work of counteracting a ‘seduction’ of the psyche into the mind.^48^

It is in precisely this light that Amis presents his toothache during 1994. In the early parts of the memoir, it is given a threefold significance. First it is a private, Oedipally charged, signifier of the fragility of family relationships. Second, it is a source of shame, leading him to recoil from others. Finally, as the examples with his aunt and Lamorna Seale show, it supplies a means of evading the knowledge of other people’s suffering. Becoming aware of each of these associations enables him to initiate the deep psychic change on which the first part of the book depends.

My aunt’s visit set me thinking, if that is quite the word I want, about the unassimilably dismal event of the previous December. Can you think about something you can’t assimilate? I don’t think you can.^49^

I suggest that he assimilates it by relinquishing his sense of safety through a series of childhood memories. He recalls that when he was at school he was regularly made to prepare for a nuclear strike by the Soviet Union. He had to lie down on the floor and hope that his desk lid would protect him from the end of the world. He imagines what it must have been like for David Partington, Lucy’s brother to find that she had not come home from Cheltenham on the night he drove her there. David and Lucy were in his mind when he wrote in the ‘Preface’ to *Einstein’s Monsters* (1987) that ‘[nuclear weapons] make me feel as if a child of mine has been out too long, much too long, and already it is getting dark’.^50^ He also recalls Kingley’s unsympathetic response to his hatred of nuclear weapons.

My brother Philip does a flawless imitation of Kingsley in this state: the whole head vibrating, the eyes dangerously swollen, the tensed mouth in a violent false smile, and (most tellingly) the nails of the forefingers scrabbling, almost bloodily, at the cuticles of the thumbs … ^51^

Arriving early at Lucy’s memorial service, he conducts a thought experiment. He imagines each of his sons in the situation Lucy found herself in at ‘the moment when they sensed the magnitude of the undifferentiated hatred that was ranged against them. The first time I did this I teetered back on my feet … ’ There are multiple projective identifications in this thought experiment and multiple shared body schemas. In one of them, he identifies with Fred West and projects Lucy’s fate into his sons. He identifies further with his sons in that situation and feels a sharp pang of guilt. He becomes at once the bereaved parent and the abandoned child. He identifies further with Lucy’s brother David who is described as ‘[veering] back’ when he learns that Amis has read every book he can lay his hands on about Fred West. The teetering is a sign that Amis’s new-found capacity for empathy works through his body. From this moment on, Amis will present his body as a vessel in which other people’s psychophysical experience can be reclaimed and, most crucially, transformed. He says that on the day of the memorial service, he had a toothache so bad ‘the bulge on the side of my jaw threatened closure of my right eye’. But it was still a cathartic experience.

How very badly my body needs this, as it needs food and sleep and air. Thoughts and feelings that had been trapped for twenty years were now being released. They were very ready. I have known literary catharsis and dramatic catharsis, and I have mourned and have been comforted; but I had never experienced misery and inspiration so purely combined.^52^

The lesson of 10 July 1994 seems to be that experiences can be shared. Amis becomes curious about the experience of others. It is common enough in memoirs for authors to use their own infirmities to imagine those of a loved one. If the loved person happens to be dead, the process of identifying with him or her almost always has the effect of turning the person into a sacrificial proxy: they do the life-writer’s dying for them. In Amis’s case, as we shall see, the main sacrificial proxies are Kingsley and Lucy Partington (but they are far from alone). The effect is offset by the increasing use of literary analogues.

The next two chapters are all about Amis’s confrontation with his own death. He goes to New York and sees his dentist, Mike Szabatura who finds in addition to rotten teeth ‘a ridge of darkness just above the chin. This, I learned, could be one of three things: a cancerous growth; a growth with a long name that would keep coming back; a growth, but something manageable and unexotic’.^53^ Although Amis does not say so, the survival prospects for someone with oral cancer were not good in 1994. For the next several chapters, the reader has to wonder if he is reading a memoir by a dying man. It is in this chapter that literary characters and literary events become a factor in Amis’s imaginative relationships with others. Around the time of Lucy Partington’s memorial service, Amis goes to Long Island with Isabel Fonseca for a holiday at her mother’s house. There, he reads aloud to Isabel’s dying brother, Bruno, whom Amis likens to ‘Eliot’s Christ, an infinitely gentle, infinitely suffering thing’. He mentions two of the stories that he read to Bruno: Borges’ *The Circular Ruins* and Kafka’s *The Fasting-Artist*, noting ruefully that ‘every paragraph that left my lips seemed to be a sinisterly poetic commentary on his condition’. The first is about a dying man who hopes to dream a man into existence, ‘modelling’ him as he dreams. ‘All that weekend,’ Amis writes, ‘I was a model of calm … I felt modeled’.^54^ The second is about a performer who after a sequence of misfortunes hires himself out to a circus where he dies of starvation, unnoticed. Amis quotes the final paragraph of the story:

they buried the fasting-artist together with the straw. Into the cage they now put a young panther. It was a palpable relief even to the most stolid to see this savage animal thrashing about in the cafe that had been bleakly lifeless for so long. He lacked nothing. The food he liked was brought to him by his keepers without a second thought; even freedom he did not appear to miss; that noble body, endowed almost to bursting-point with all it required, seemed to carry its very freedom around with it – somewhere in the teeth apparently; and sheer delight at being alive made such a torch of the beast’s breath that the spectators had difficulty in holding their ground against it.^55^

I am suggesting that in both cases Amis uses his identifications with figures in the stories as counterweights enabling him to survive his identification with Bruno. Bruno bears the brunt of Amis’s mortality (attributive projective identification) while Amis appropriates the vitality of the modelled man and the panther (acquisitive projective identification).

This hardens into something of a pattern in the chapter dealing with his maxillofacial surgery, entitled ‘Him Who is! Him Who Was!’ The chapter begins with a quotation from a poem in Donne’s *Songs and Sonets*, ‘A Nocturnall upon *S. Lucies* day, Being the shortest day’. The speaker mourns his beloved and claims to be the emblem of ‘every dead thing, / In whom Love wrought new alchemy’. The connection between the ‘S. Lucie’ of Donne’s poem and Lucy Partington is reinforced by the names and Amis notes that Lucy disappeared on 27 December, close to the shortest day. Amis quotes the ending of the poem in which the poet expresses a wish to join his beloved in heaven.

Since shee enjoyes her long nights festivall,
Let mee prepare towards her, and let mee call
This houre her Vigill, and her eve, since this
Both the yeares, and the dayes deep midnight is.^56^

Amis immediately ‘prepares towards’ *his* Lucy when he presents himself for a CAT scan. Lucy’s body had been ‘decapitated and dismembered, and her remains were crammed into a shaft between leaking sewage pipes, along with a knife, a rope, a section of masking tape and two hair-grips’.^57^

With an emery board between my jaws, a blue showercap on my head, with straps over my brow and chin, I was sucked backwards into a kind of cyclotron where I remained for ten minutes. The confinement or internment made me think helplessly about Lucy Partington.^58^

He appropriates *his* Lucy’s death and long interment in order to reduce the threat of what the CAT scanner might do or reveal. Kingsley’s claustrophobia serves a similar purpose. Amis remembers how for a time during his twenties he became frightened of travelling on the underground, a fear shared by his father. Here, the comparison is terrifying but not fatal: the train going through the underground tunnel is like the couch on which the patient lies that slides through the CAT scanner. Donne’s poem enables Amis to commemorate his cousin and to face his own mortality ceremoniously. Concomitantly, Lucy and Kingsley supply templates through which worries about his own fate can be diminished. The same pattern of attributive and acquisitive projective identifications is in play. Lucy and Kingsley can bear the burden of his mortality for him. He meanwhile ‘prepares towards’ them, ignorant of when he will actually die.

The remainder of the chapter focuses on Joyce, Nabokov and Yeats. Joyce and Nabokov lost their teeth and wrote novels whose protagonists suffer dental agonies no less severe than Amis’s own. Stephen Dedalus, Joyce’s fictional alter ego, thinks to himself that his teeth have turned to shells. He calls himself ‘toothless Kinch, the superman’. Joyce’s poem ‘A Prayer’ was written in 1924 after an eye operation which left him blind in one eye. Amis notes that while the poem is generally assumed to be addressed to Joyce’s wife, when he thinks of the poem he thinks of his dentist.

‘The second of the three stanzas runs (beautifully and to me unbearably)’:
I dare not withstand the cold touch that I dread
Draw from me still
My slow life! Bend deeper on me, threatening head,
Proud by my downfall, remembering, pitying
Him who is, him who was!’^59^

The basic elements of Donne’s poem – two lovers and death – are repeated here; but in Joyce’s poem the beloved holds sway over death because she is somehow in league with it. Amis notes Richard Ellmann’s gloss that the poet ‘confuses desire and pain, [pain] because his mind associates his subjection to this beloved with other subjections – to eye trouble and to death.’ But he does not quote Ellmann’s further comment that ‘As he exerted total control over his books, Joyce dreamed of agonizing self-abandonment to female power’.^60^ This observation is surely relevant to Amis’s theme. For if his dentist Mike Szabatura is the allegorical figure of death drawing Amis’s slow life from him (you could say he becomes Amis’s very own Fred West), Isabel is his Nora Joyce.

One of the most interesting literary digressions concerns Nabokov’s campus novel, *Pnin* (1957). The eponymous hero of that book has poor teeth and a heart condition when he arrives at a small East Coast liberal arts college to take up a professorship of Russian. Two events are given special significance in Amis’s summary. First, Pnin has to have his teeth replaced by dentures. Second, he receives a letter from his ex-wife, Liza, the love of his life, who pretends she would like to marry him again. The removal of the teeth is recorded in full:

His tongue, a fat sleek seal, used to flop and slide so happily among the familiar rocks, checking the contours of a battered but still secure kingdom, plunging from cave to cove, climbing this jag, nuzzling that notch, finding a shred of sweet seaweed in the same old cleft; but now not a landmark remained and all there existed was a great dark wound, a terra incognita of gums which dread and disgust forbade one to investigate.^61^

Shortly after this ordeal, he discovers that Liza’s overture was a mere ruse to make him pay for her passage to America. Pnin is unconsolable. ‘I haf nofing left, nofing, nofing!’Amis quotes these lines in order to reveal a further motive for not having his teeth removed. ‘I sometimes believed that sex and teeth would be coterminous,’ he writes. ‘Love would end. Poor Pnin had nothing.’ But in Amis’s case, Isabel Fonseca

came bellydancing out of the bathroom wearing (a) your silk bathrobe and (b) my teeth. Both were then removed.

This was the war against shame.

The next morning I woke early and lay there quietly laughing and weeping into the pillow. I felt fragile, guileless and exquisitely consoled. The quality of the happiness made me think of a poem – early Yeats – that I had once copied out for my sister to memorise, thirty years ago. Had I the … the dark cloths of light and night and half night … I have spread my … Tread softly because you … ^62^

The rehearsal of the workings of a tired mind with ellipses is surely a sly tribute to the ‘Eumaeus’ chapter of *Ulysses*. And the point about the Yeats poem that he remembers (‘He Wishes for the Cloths of Heaven’) is that like ‘A Prayer’ it is about submission to the beloved but with the important difference that in it, happy vulnerability replaces death. By wearing Amis’s dentures, Isabel Fonseca transforms the symbolic equation of teeth with mortality. Death has been conquered and all of the tragic associations of teeth are temporarily subsumed into Eros.

Amis couches his autobiography as the history of his relationship to his body. How should we receive it? Is it a credible account of an embodied life or merely a novelist’s ‘Just So’ story? Roger Luckhurst inclines to the latter view. He argues that since the 1990s, writers of commercially successful memoirs have had to demonstrate inwardness with trauma. Trauma has acquired the status of the true sign of the real. The fate of Lucy Partington enabled Amis to lay claim to credibility in these terms.^63^ A number of critics have complained that Lucy Partington became a much more significant figure for Amis through the writing of *Experience* than she had ever been when she was alive. Marian Partington, Lucy’s sister, said he hardly knew her.^64^ And how heavily did Kingsley’s fate really weigh on him? Again, the same critics note that he chose not to be present at Kingsley’s death.

Although these criticisms may be true, they miss their target. For Amis is quite explicit about his use of other people in the memoir. As I have already stated, people are first and foremost *bodies* here. We are bound to them by our shared physical experiences. It is by investigating the hidden aspects of their physical experience that we encounter their humanity. The differentiation of these three kinds of inter-relatedness dominates the first part of the memoir. In the first place, there are experiences that threaten to overwhelm him. The bottomless adolescent *cafard*, the mincing, the tramp dread, the shame at being small or otherwise repellent to women, the dental reverie he falls into in the presence of his aunt nine months after the disappearance of her daughter, fall squarely into this category. These are distinct from a second category comprising much rarer moments of relatively unburdened engagement with an expanding world. Under this heading we might number his becoming ‘Osric’ in Brighton/Elsinore, ‘in love with poetry’, and his two experiences of receiving Robert Graves’s hospitality. Finally, there is a third category of bodily experiences that are shared that enable him to develop. When he sees himself as a version of Rafael, the ‘flailing blur’, he can accept himself. Amis tells us that after Elizabeth Jane Howard left Kingsley, he used to numb himself to sleep with ‘late night carbo-fests’: ‘As if in the interests of hibernation he would load up his cheeks with confectionery at about twice the rate that he ingested it’.^65^ This indulgence creates an uncanny parallel with Amis in his postoperative state. ‘Jesus, Dad’, I once said, ‘what’s going on in there? Your face is the size of a basketball’.^66^ It is as if all his life he has unwittingly been recapitulating Kingsley’s psycho-physical trajectory. Superficially, this memory supplies uncanny retrospective confirmation to Amis that ‘grief lives in the mouth’. But perhaps more importantly it teaches him that bodily sensation is continuous with some very archaic areas of mentalisation. There is a kind of grief that can only be apprehended through bizarre physical experience.

Recognition of others repeatedly takes the form of identifications with *their* physical experience. David’s jolt on learning that Amis has read ‘all the books’ about Fred West; Kingsley’s binge-eating of cakes and sweets after the breakup of his second marriage; Hilary’s looking at her toothless reflection in a mirror, but above all, Lucy’s murder and abandonment become psychophysical precedents or parameters enabling Amis to submit to major maxillofacial surgery and to change his life. The claim that these precursor experiences seem to make cumulatively (though it is never made explicitly) is that rebirth of the mind is only possible through the rebirth of the body. I suggested earlier that the consciousness of another person’s suffering is invisibly present in the first two chapters which take the form of Amis talking to his sons while driving a car. These car journeys are initiated by a driver who has been transformed by the knowledge of what happened after Lucy Partington’s last-known car journey (when she was driven to Cheltenham by her brother David). The antithesis between this knowledge and his sons’ innocence could hardly be greater. With great skill, the reader is put in his sons’ place early in the memoir.

I am not suggesting that is Amis is knowingly engaging with post-Kleinian psychoanalytic thinking. But I do think that the developmental story he puts forward about his bodily experience is strikingly consonant with a theory offered by Armando Ferrari (1922–2006). In his book *From the Eclipse of the Body to the Dawn of Thought* (2004), Ferrari argues that our first object relationship is not with the breast or the mother but with the body.^67^ The body radiates sensation. Ferrari calls it the ‘Concrete Original Object’ because we are always drawn back to it. It ‘stands before the mind as a permanently unsolved problem’. The task of other objects – parents and carers – is to help the infant to bear his relationship with his body. Ferrari sees development taking place along two perpendicular axes. The vertical or ‘Onefold’ axis encapsulates the infant’s (and later the individual’s) relationship with his body. The horizontal or ‘Twofold’ axis governs his relationship with others. The mind ‘cools down’ the heat emanating from the body.^68^ Eventually, it manages to throw it under eclipse, though never permanently. Ferrari’s extension of Bion’s theory arises from his insistence that the *body* needs another person’s reverie as much as the mind. I think the imaginative faculty on which Amis lays so much stress when thinking about the affection in which Rafael is held is a version of this somatically inflected reverie.

Another Italian psychoanalyst influenced by Bion, Antonino Ferro (1947–), has argued that psychosomatic disorders are signs of facts that have gone unprocessed not only by the patient but also by those he loves.^69^ Once these facts are ‘seen’ it becomes possible to isolate the psychosomatic disorder as a medical problem. What seems to happen in *Experience* is the author stumbles upon the discovery that mental and physical ills must be ‘seen’ and experienced collectively.

It might be objected that Amis is creating his own personal psychosomatic mythology and that none of the claims I am extrapolating from his book have any wider significance. The only way to test this is for scholars of life-writing and illness narrative to collect a range of testimonies from writers who have tried to reconstruct their experience in ways that pay close attention to the body. Here I would enter the caveat that only writers of great skill can capture the interplay of the mind and the body in the way that Amis does in *Experience*. But I see no reason why a range of equally skilled narratives would not help to enrich our sense of the body and its contribution to selfhood more generally. Amis writes from a strongly male and able-bodied perspective. It is likely that narratives of the body by women would yield further and different information about the interplay of the body and the mind. I would contend that first-person memoirs offer theorists of life-writing and others the opportunity to challenge the anti-mental bias of modern biomedicine by reclaiming the whole experiential field in which illness occurs in the West today. The turn to complex neurological theory by some life-writing theorists in attempting to understand mind-body relationships is based on an undue degree of pessimism concerning the power of creative writers to describe their own and our experience authoritatively.^70^ I hope that this paper has supplied grounds for exploring a different approach.

## Notes

1. M. Amis, *Experience: A Memoir* (London: Talk Miramax Books, 2000).

2. G. Keulks, *Father and Son: Kingsley Amis, Martin Amis, and the British Novel Since 1950* (Madison: University of Wisconsin Press, 2003), pp. 199–219 and N. Powell, *Amis and Son* (London: Macmillan, 2008).

3. Amis, *Experience*, p. 80.

4. Martin Amis, *The Pregnant Widow* (London: Cape, 2011). The epigraph from Ovid is taken from Ted Hughes' *Tales From Ovid* (London: Faber, 1997), p. 3.

5. John Banville, ‘Executioner Songs’, *New York Review of Books*, 1 March 2007. http://www.nybooks.com/articles/archives/2007/mar/01/executioner-songs/

6. Winnicott first used this term in a review of Carl Jung's autobiography. D.W. Winnicott, ‘Memories, Dreams, Reflections: By C. G. Jung’, *International Journal of Psycho-Analysis* 45 (1964), pp. 450–5.

7. P. Tew, ‘Alexithymia and a Broken Plastic Umbrella: Contemporary Culture and Martin Amis's Money’, *Textual Practice*, 26.1 (2012), pp. 99–114.

8. The term alexithymia was coined by Peter Sifneos, a Harvard psychiatrist and psychoanalyst specialising in the treatment of psychosomatic disorders. P. E. Sifneos, ‘The Prevalence of “Alexithymic” Characteristics in Psychosomatic Patients’, *Psychotherapy and Psychosomatics*, 22.2 (1973), pp. 255–62.

9. Tew, ‘Alexithymia’, p. 104.

10. M. Amis, *Money: A Suicide Note* (London: Cape, 1985), p. 14.

11. Amis, *Experience*, p. 51.

12. Ibid., p. 53.

13. Amis, *Money*, p. 26.

14. Tew, ‘Alexithymia’, p. 112.

15. I would suggest that Amis's hostility to Islam and fierce identification with Israel post 9/11 might be traced to peculiar status of Jewishness in the memoir.

16. The idea of the body schema in fact derives from H. Head and G. Holmes, ‘Sensory Disturbances from Cerebral Lesions’, *Brain*, 34 (1911), pp. 102–254.

17. Paul Schilder, *The Image and Appearance of the Human Body* (London: Routledge, 1950), p. 166. Schilder was a neurologist, psychiatrist and psychoanalyst. He also had a PhD in phenomenology. Elizabeth Grosz used Schilder's distinction in her classic feminist study of embodiment, *Volatile Bodies: Towards a Corporeal Feminism* (Bloomington: Indiana University Press, 1994), see especially pp. 67–81.

18. M. Klein, ‘Notes on Schizoid mechanisms' in *Envy and Gratitude and Other Works*, *1946–1963* (London: The Hogarth Press, 1975), pp. 1–24.

19. This concept first appears in Klein's book, *The Psycho-Analysis of Children* (London: The Hogarth Press, 1932), p. 230.

20. M. Klein, ‘Our Adult World and Its Roots in Infancy’, in *Envy and Gratitude* (London: The Hogarth Press, 1975), pp. 251–2.

21. R. Britton, *Belief and Imagination: Explorations in Psychoanalysis* (London: Brunner-Routledge in collaboration with the Institute of Psychoanalysis, 1998), pp. 5–6.

22. W. R. Bion, *Learning from Experience* (London: William Heinemann Medical Books, 1962, repr. Karnac Books, 1984), pp. 35–6.

23. M. Merleau-Ponty, *The Phenomenology of Perception.* Trans. C. Smith. Trans. rev. F. Williams and D. Guerrière. (London: Routledge, 2003), p. 412.

24. Ibid., p. 354.

25. M. Merleau-Ponty, ‘The Child's Relations With Others’, trans. W. Cobb, in J. M. Edie (ed.), *The Primacy of Perception* (Evanston, IL: Northwestern University Press), p. 118.

26. J. Sandler, ‘The Background of Safety’, *International Journal of Psycho-Analysis* 41 (1960), pp. 352–6.

27. J. Lanchester, ‘Be Interesting!’ *London Review of Books* 22.13 (2000), pp. 3–6.

28. Ibid., p. 3.

29. Ibid., p. 14.

30. Ibid.

31. Merleau-Ponty, *Phenomenology of Perception*, p. 122.

32. Ibid., p. 125.

33. Amis, *Experience*, p. 15.

34. Ibid., pp. 35–6.

35. A. Malin and J. Grotstein, 'Projective Identificaiton in the Therapeutic Process', *International Journal of Psycho-Analysis* 47 (1966), pp. 26–42.

36. Ibid., p. 40.

37. Ibid., pp. 41–2.

38. Ibid., p. 43.

39. Ibid., p. 44.

40. Ibid.

41. Ibid., p. 45.

42. Ibid., p. 48.

43. Ibid.

44. Ibid.

45. Ibid., p. 54.

46. D. W. Winnicott, ‘Mind and Its Relation to the Psyche-Soma’, in *Through Pediatrics to Psychoanalysis* (London: The Hogarth Press and the Institute of Psychoanalysis, 1975), pp. 243–55.

47. Ibid., p. 243.

48. Ibid., p. 253.

49. Amis, *Experience*, p. 52.

50. Ibid., p. 60.

51. Ibid., p. 59.

52. Ibid., pp. 69–70.

53. Ibid., p. 77.

54. Ibid., p. 79.

55. Ibid.

56. Ibid., p. 110.

57. Ibid., p. 62.

58. Ibid., p. 112.

59. Ibid., p. 115.

60. R. Ellmann, *James Joyce* (Oxford: Oxford University Press, 1959, corrected edition 1983), p. 565.

61. Amis, *Experience*, p. 116.

62. Ibid., p. 125.

63. R. Luckhurst, *The Trauma Question* (London: Routledge, 2008), p. 117. But I think that Amis's focus on these visceral and more primitive sources of personhood which he shows to be relatively indifferent to relating to whole persons indicates his distance from ‘trauma culture’.

64. See, for example, R. Brook, ‘Amis Exploited West Link’, *Sunday Times*, 11 June 2000.

65. Ibid., p. 93.

66. Ibid., p. 94.

67. A. B. Ferrari, *From the Eclipse of the Body to the Dawn of Thought*, trans. I. Chigi (London: Free Association Books, 2004).

68. Ibid., p. 38–9.

69. A. Ferro, *Mind Works: Technique and Creativity in Psychoanalysis*, trans. P. Slotkin (London: Routledge, 2009), p. 90.

70. See, for example, P. J. Eakin, *How Our Lives Become Stories* (Ithaca, NY: Cornell University Press, 1999).

